# Factors affecting titanium mesh cage subsidence in single-level anterior cervical corpectomy and fusion for ossification of the posterior longitudinal ligament

**DOI:** 10.1186/s13018-022-03409-6

**Published:** 2022-12-01

**Authors:** Yifan Tang, Xiangwu Geng, Fengning Li, Yanqing Sun, Lianshun Jia, Shengyuan Zhou, Xiongsheng Chen

**Affiliations:** Department of Orthopedics, Spine Center, Shanghai Changzheng Hospital, Second Military Medical University, 415 Fengyang Road, Huangpu District, Shanghai, China

**Keywords:** Titanium mesh cage subsidence, Anterior cervical corpectomy and fusion, Ossification of the posterior longitudinal ligament, Distraction degree, Cervical curvature

## Abstract

**Purpose:**

To analyze risk factors of titanium mesh cage (TMC) subsidence in single-level anterior cervical corpectomy and fusion (ACCF) for cervical ossification of the posterior longitudinal ligament (OPLL).

**Methods:**

TMC subsidence is defined as the reduction of the adjacent vertebral bodies by ≥ 2 mm. Patients with cervical OPLL who were treated with single-level ACCF between January 2019 and May 2021 were retrospectively analyzed in two groups: patients with TMC subsidence as Group S and patients with no TMC subsidence as Group N during the one-year follow-up period. The degree of distraction of surgical segment and correction of the cervical curvature was measured to analyze their relationship with TMC subsidence.

**Results:**

A total of 128 patients were included in Group S, and 138 patients were included in Group N. There was no significant difference in patient demographics and complications between the two groups. The degree of distraction in Group S was significantly higher than that in Group N (11.4% ± 7.6% vs*.* 4.7% ± 9.7%, *P* < 0.01). The change of C2 to C7 Cobb angle (*α*) in Group S was significantly greater than that in Group N (5.7 ± 2.7 vs*.* 1.4 ± 4.7, *P* < 0.01), and the change of interspinous process distance (SPD) in Group S was also significantly greater than that in Group N (7.0 ± 4.2 vs. 4.1 ± 2.7, *P* < 0.01). The JOA score and JOA recovery rate were not statistically different between the two groups.

**Conclusions:**

Intraoperative selection of overlength TMC in single-level ACCF for OPLL, over-distraction and excessive correction of the cervical curvature may cause TMC subsidence after surgery. No significant impact of TMC subsidence on the surgical outcome was observed during the 1-year follow-up period.

## Introduction

Anterior cervical corpectomy and fusion (ACCF) by titanium mesh cage (TMC) placement is an effective procedure for the treatment of cervical spine diseases, including cervical ossification of the posterior longitudinal ligament, cervical spondylosis, cervical spine hyperextension injury and tumors. ACCF can rebuild the stability of the cervical spine by directly decompressing the compressed spinal cord, thus providing patients with long-term improvement (1–4). Autologous iliac bone grafting is the gold standard for reconstruction of bone defects in corpectomy. However, about 25% patients may develop donor site complications, including local pain, infection and hematoma formation at the donor site (5–7). The use of TMC can avoid the occurrence of donor site complications and establish early biomechanical stability (3). However, there is a high risk of TMC subsidence ranging from 9% to 79.7% according to different definitions (8–11). In this article, we retrospectively analyzed the data of 68 patients with ossification of the posterior longitudinal ligament after ACCF, TMC and titanium plate internal fixation from January 2019 to May 2021 and explored the relationship between TMC subsidence and over-distraction and excessive correction of the cervical curvature.

## Materials and methods

### Medical records

This study retrospectively analyzed patients who were treated with ACCF, TMC and titanium plate internal fixation for cervical OPLL from January 2019 to May 2021 in the Spine Surgery Center of Shanghai Changzheng Hospital (Shanghai, China). The inclusion criteria were patients (1) who were diagnosed with cervical ossification of the posterior longitudinal ligament through X-ray, MRI and CT scan before surgery; (2) who received single-level ACCF, TMC and titanium plate internal fixation because of the presence of local compression on the spinal cord; and (3) who were followed up consecutively at 1 day, 3 months, 6 months and 12 months after operation. The exclusion criteria were patients with (1) simple intervertebral disk degeneration; (2) a history of trauma, infection, tumor, osteoporosis or other serious neurological diseases; (3) incomplete follow-up data; and 4) a history of substance abuse.

### Surgical procedures and postoperative treatment

The patient was placed in a supine position, and a transverse incision was made on the front right side of the neck. The skin and subcutaneous tissue were cut in sequence, the subcutaneous space was freed, the platysma was cut longitudinally, and the joint fascia was cut. After retracting the trachea and esophagus to the left, the anterior fascia was cut to expose the vertebral body and the front of the intervertebral disk. The intervertebral space was propped up by using a distraction screw in the vertebral body. After removing the intervertebral disk, the vertebral body was subtotally removed, and the endplates at both ends were completely retained. At the same time, the patients were treated with 500 mg methylprednisolone. An appropriately sized (*d* = 1.0 cm) titanium mesh cage (TMC) prefilled with the local bone harvested from corpectomy was placed into the decompressed area between the endplates and tightly hammered into place. After removing the distraction nail, a prebent titanium plate of an appropriate length was fixed on the upper and lower vertebral bodies of the TMC with non-constrained screws. The position of the inner plant was confirmed by X-ray. After achieving hemostasis in the incision, a drainage tube was placed, and the incision was sutured layer by layer.

Cefuroxime or clindamycin was used to prevent infection. In the first three days after surgery, the patients were treated with 120 mg, 80 mg, or 40 mg methylprednisolone. Nebulized inhalation was applied to reduce airway reactions. After 24 h, the patients advised to begin ambulation under the protection of a Philadelphia collar, and the positions of the TMC, titanium plates and screws were evaluated and confirmed by cervical X-ray radiography.

### Clinical evaluation

**Fig. 1:**
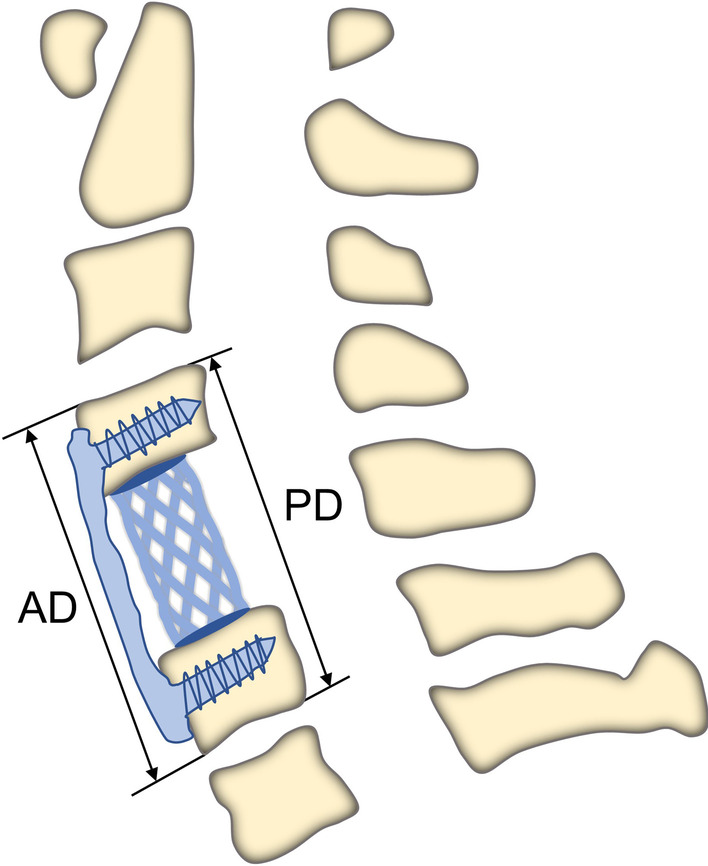
Measurement of TMC subsidence. *AD* anterior distance. *PD* posterior distance

**Fig. 2:**
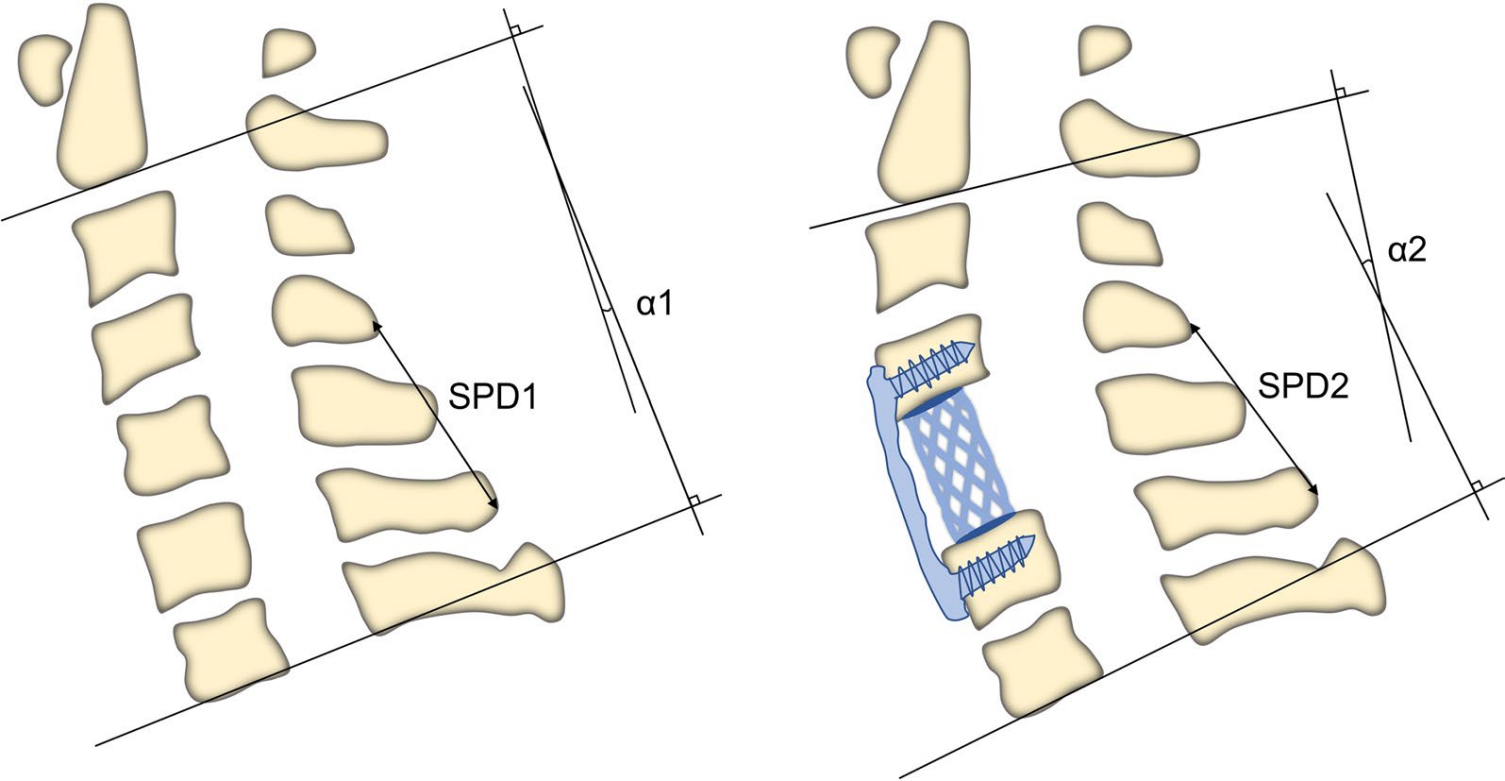
Correction of the cervical curvature. The degree of recovery of cervical lordosis: *α* = *α*2–*α*1. The change of spinous process distance: SPD = SPD1–SPD2

The incidence of TMC subsidence was assessed by cervical spine X-ray films during the 1-year follow-up period at 1 day, 3 months, 6 months and 12 months after surgery. TMC subsidence is defined as the reduction of the distance between the upper and lower vertebral bodies on the lateral X-ray film of the cervical spine, the anterior height (AD) or posterior height (PD) of the adjacent vertebral bodies by ≥ 2 mm (Fig. 1). The method of judging bone fusion is plain radiography showing the formation of a mature trabecular bridge between the TMC and the adjacent endplates. The change of C2 to C7 Cobb angle (α) and the change of the interspinous process distance (SPD) of the fusion segments were measured on one-day post-operation to assess the degree of correction of the cervical spine curvature (Fig. 2). The larger the *α* is, or the smaller the SPD is, the greater the correction of curvature is. The relationship between excessive distraction and TMC subsidence was analyzed by calculating the difference between the pre- and postoperative expansion height using the following equation: degree of distraction = [(postoperative AD) − (preoperative AD)]/(preoperative AD) × 100%. Before surgery and during the follow-up period, neurological function was evaluated independently by different physicians using the Japanese Orthopedic Association (JOA) score system. The JOA recovery rate = (postoperative JOA-preoperative JOA)/(17-preoperative JOA) × 100%.

### Statistical analysis

Statistical analysis was performed by using SPSS 22 (SPSS Inc, Chicago, IL). *X*_*2*_ test was used to compare the patient’s gender, age, operation segment and complication rate. Differences in JOA score, JOA recovery rate, *α*, SPD, degree of distraction, operation time and intraoperative blood loss between groups were analyzed by *t* test. Repeated measurement data within the groups were tested by Hotelling T2 test. *P* < 0.05 was considered statistically different.

## Results

### Characteristics of the patients

A total of 266 consecutive patients were finally included in this study, including 128 patients with TMC subsidence (Group S) and 138 patients with no TMC subsidence (Group N). The general information of the patients is shown in Table 1. There were no significant differences in age, gender, operation segment, operation time or intraoperative blood loss between the two groups (*P* > 0.05).

**Table 1:** Characteristics of the patients

	Group S	Group N	*P* value
Number of patients	128	138	
Age (yr)	58.3 ± 7.2	57.4 ± 7.5	0.34
Sex			
Male	80	108	0.07
Female	48	30	
Level of surgery			
C4	32	72	
C5	64	42	
C6	32	24	
Operation time (min)	78.4 ± 10.8	76.6 ± 12.1	0.37
Intraoperative blood loss (ml)	149.2 ± 30.8	139.5 ± 30.5	0.07
Number of CSFL cases	14 (10.9%)	8 (5.80%)	0.18
Number of C5 palsy cases	4 (3.1%)	2 (1.4%)	0.43

Group S: TMC subsidence group

Group N: TMC non-subsidence group

CSFL: cerebrospinal fluid leakage

### The relationship between TMC length, intraoperative over-distraction or cervical curvature overcorrection and TMC subsidence

The results showed that the mean length of TMC in Group S was longer than that in Group N (31.5 ± 4.5 mm vs. 27.5 ± 3.9 mm, *P* < 0.01). The degree of distraction in Group S was significantly higher than that in Group N (11.4% ± 7.6% vs*.* 4.7% ± 9.7%, *P* < 0.01), indicating that excessive distraction during the operation or too long TMC may cause postoperative TMC subsidence. The α in Group S was significantly greater than that in Group N (*P* < 0.01), and SPD in Group S was significantly greater than that in Group N (*P* < 0.01) (Table 2), indicating that overcorrection of the cervical spine curvature could also lead to TMC subsidence.

**Table 2:** Possible reasons for TMC subsidence

	Group S	Group N	*P* value
*L* (mm)	31.5 ± 4.5	27.5 ± 3.9	< 0.01
Degree of distraction (%)	11.4 ± 7.6	4.7 ± 9.7	< 0.01
*α* (°)	5.7 ± 2.7	1.4 ± 4.7	< 0.01
SPD (mm)	7.0 ± 4.2	4.1 ± 2.7	< 0.01

Group S: TMC subsidence group

Group N: TMC non-subsidence group

L: TMC length

SPD: spinous process distance

### The relationship between TMC subsidence and surgical efficacy

In Group S, the mean JOA score at 1 day after operation was 13.7 ± 1.4, and the JOA recovery rate was 37.8% ± 21.3%; the mean JOA score at final follow-up was 14.2 ± 1.5, and the JOA recovery rate was 45.3% ± 25.3%. In Group N, the mean JOA score was 14.0 ± 1.5 on the first postoperative day, and the JOA recovery rate was 35.2% ± 25.8%; the mean JOA score at the last follow-up was 14.5 ± 1.6, and the JOA recovery rate was 46.8% ± 25.8%. All these postoperative data between Group S and Group N were not significantly different (*P* > 0.05) (Table 3).

**Table 3:** JOA score and JOA recovery rate during the 1-year follow-up period

	Group S	Group N	*P* value
JOA score			
Preoperative	11.6 ± 1.5	12.0 ± 1.7	0.05
1-day postoperative	13.7 ± 1.4	14.0 ± 1.5	0.07
Final follow-up	14.2 ± 1.5	14.5 ± 1.6	0.09
JOA recovery rate (%)			
1-day postoperative	37.8 ± 21.3	35.2 ± 25.8	0.38
Final follow-up	45.3 ± 25.3	46.8 ± 25.8	0.62

Group S: TMC subsidence group

Group N: TMC non-subsidence group

JOA: Japanese Orthopedics Association

### The relationship between TMC subsidence and surgical complications

Cerebrospinal fluid leakage (CSFL) occurred in 7 (10.9%) cases in Group S. In Group N, CSFL occurred in 4 (5.80%) cases. C5 nerve palsy occurred in 2 (3.1%) cases in Group S and one (1.4%) case in Group N (Table 1). All these postoperative complications in the above 28 cases were cured after non-surgical treatment and rehabilitation exercises. There was no statistical difference in the incidence of complications between the two groups (*P* > 0.05).

## Discussion

Surgical treatment strategies for OPLL include anterior, posterior and anteroposterior approaches (12–14). The advantage of anterior surgery lies in that it can remove the ossified mass and achieve direct decompression. After anterior decompression, the bone graft or internal plants including the titanium plates, screws, cage and TMC are usually needed to reconstruct the anatomical integrity and stability of spine and achieve bone fusion (15, 16). ACCF is a commonly used anterior procedure for the treatment of OPLL, especially for decompression of the compression behind the vertebral body, which cannot be achieved by ACDF (17–19). Since the advent of TMC in 1986, it has been widely used to support the anterior spine and restore the natural alignment of the cervical spine (20). At present, there are multiple TMC designs for clinical use, including application of the endcaps at both ends of the TMC to increase the contact area between the TMC and the adjacent endplates, thereby preventing TMC subsidence (21–23). However, the incidence of TMC subsidence still fluctuates between 9% and 79.7% despite various designs to optimize TMC (8–11). The result of our study showed that the incidence of TMC subsidence incidence was 48.1% in single-level ACCF for OPLL during the 1-year follow-up period. Since patients with osteoporosis were excluded in our study, this may exclude most cases of TMC subsidence. At the same time, we defined TMC subsidence as the reduction of AD or PD of the adjacent vertebral bodies ≥ 2 mm, while some researchers also included TMC subsidence 1–3 mm in their studies(8).

To the best of our knowledge, no study has confirmed whether there is an association between the TMC length and TMC subsidence. Our results showed that the TMC length in Group S was significantly greater than that in Group N, and the distraction degree in Group S was also significantly greater than that in Group N, suggesting that an excessive length of TMC and excessive distraction of the decompression segments may be related to TMC subsidence after surgery. In addition, our research also showed that both α and SPD in Group S were significantly greater than those in Group N, indicating that intraoperative curvature was over-corrected. The greater the degree of cervical lordosis after surgery may also be a relevant factor of the occurrence of TMC subsidence. Studies have shown that with the distraction height increasing, the load between the TMC and the vertebral body may exceed the bearing capacity of the vertebral body, resulting in TMC subsidence (24). However, the insufficient distraction height is difficult to restore the normal cervical curvature and intervertebral height. At present, there is no specific standard for the distraction height reported in the literature.

Our results showed that the JOA score and JOA recovery rate after ACCF for OPLL were significantly increased, indicating that ACCF is an effective surgical treatment for OPLL, which is consistent with existing reports in the literature (25, 26). However, we found no significant difference in JOA score and JOA recovery rate between Group S and N during the 1-year follow-up period, indicating that there is no significant correlation between TMC subsidence and surgical efficacy. It has been reported in the literature that cage subsidence after anterior cervical surgery can lead to new neck pain and radiculopathy, and imaging examination confirmed that the intervertebral height decreased after cage subsidence (27). Anatomically, TMC subsidence could also cause the loss of intervertebral height, resulting in a reduction in the volume of the intervertebral foramen, and nerve root compression may cause new postoperative radiculopathy. However, no similar cases were observed in our series. Only 14 cases of CSFL and 4 cases of C5 palsy occurred in Group S, which were common complications after anterior cervical surgery for OPLL (28). Also, we found no significant correlation between the occurrence of complications and TMC subsidence.

In addition, we chose non-constrained screws (variable screws) during the operation to increase the fusion rate and avoid the occurrence of broken nails. However, mini-displacement of the variable screws may also cause TMC subsidence, though no related information has been reported in the literature, suggesting that design of a new screw type may be required to solve this problem.

This study has some limitations. First, this is a retrospective study that only included patients treated in our department. In addition, we only selected cases treated by the same surgeon to control the impact of individual differences on the surgical outcome. Finally, we only followed up the patients for a year after surgery.

## Conclusion

Intraoperative selection of overlength TMC in single-level ACCF for OPLL, over-distraction and excessive correction of cervical spine curvature may cause TMC subsidence after surgery. The surgical outcome was not affected by TMC subsidence during a 1-year follow-up period in our series.

## Data Availability

The raw data used and/or analyzed in this study are available from the corresponding author upon reasonable request.

## Abbreviations

TMC: Titanium mesh cage
ACCF: Anterior cervical corpectomy and fusion
OPLL: Ossification of the posterior longitudinal ligament
AD: Anterior height
PD: Posterior height
SPD: Interspinous process distance
JOA: The Japanese Orthopedic Association
CSFL: Cerebrospinal fluid leakage
